# Simultaneous exercise stress cardiac magnetic resonance and cardiopulmonary exercise testing to elucidate the Fick components of aerobic exercise capacity: a feasibility and reproducibility study and pilot study in hematologic cancer survivors

**DOI:** 10.1186/s40959-023-00182-1

**Published:** 2023-07-10

**Authors:** Justin M. Canada, John McCarty, Jennifer H. Jordan, Cory R. Trankle, Kevin DeCamp, Josh D. West, Mary Ann Reynolds, Rachel Myers, Katey Sweat, Virginia McGhee, Ross Arena, Antonio Abbate, W. Gregory Hundley

**Affiliations:** 1 VCU Pauley Heart Center, Virginia Commonwealth University, 1200 E. Broad Street, P.O. Box 980335, Richmond, VA 23298 USA; 2 Division of Hematology, Oncology & Palliative Care, VCU Massey Cancer Center, Virginia Commonwealth University, Richmond, VA USA; 3 Department of Biomedical Engineering, Virginia Commonwealth University, Richmond, VA USA; 4 Department of Radiology, Virginia Commonwealth University, Richmond, VA USA; 5 Department of Physical Therapy, College of Applied Health Sciences, University of Illinois at Chicago, Chicago, IL USA; 6 Berne Cardiovascular Research Center, Department of Medicine, University of Virginia, Charlottesville, VA USA

**Keywords:** Hematologic malignancy, Exercise stress cardiac magnetic resonance; oxygen consumption, Exercise cardiac index, Arteriovenous oxygen content difference

## Abstract

**Background:**

Patients treated for hematologic malignancy often experience reduced exercise capacity and increased fatigue; however whether this reduction is related to cardiac dysfunction or impairment of skeletal muscle oxygen extraction during activity is unknown. Cardiopulmonary exercise testing (CPET) coupled with stress cardiac magnetic resonance (ExeCMR), may provide a noninvasive method to identify the abnormalities of cardiac function or skeletal muscle oxygen extraction. This study was performed to determine the feasibility and reproducibility of a ExeCMR + CPET technique to measure the Fick components of peak oxygen consumption (VO_2_) and pilot its discriminatory potential in hematologic cancer patients experiencing fatigue.

**Methods:**

We studied 16 individuals undergoing ExeCMR to determine exercise cardiac reserve with simultaneous measures of VO_2_. The arteriovenous oxygen content difference (a-vO_2_diff) was calculated as the quotient of VO_2_/cardiac index (CI). Repeatability in measurements of peak VO_2_, CI, and a-vO_2_diff was assessed in seven healthy controls. Finally, we measured the Fick determinants of peak VO_2_ in hematologic cancer survivors with fatigue (n = 6) and compared them to age/gender-matched healthy controls (n = 6).

**Results:**

Study procedures were successfully completed without any adverse events in all subjects (N = 16, 100%). The protocol demonstrated good-excellent test-retest reproducibility for peak VO_2_ (intraclass correlation coefficient [ICC] = 0.992 [95%CI:0.955–0.999]; *P* < 0.001), peak CI (ICC = 0.970 [95%CI:0.838–0.995]; *P* < 0.001), and a-vO_2_diff (ICC = 0.953 [95%CI:0.744–0.992]; *P* < 0.001). Hematologic cancer survivors with fatigue demonstrated a significantly lower peak VO_2_ (17.1 [13.5–23.5] vs. 26.0 [19.7–29.5] mL·kg^-1^·min^-1^, *P* = 0.026) and lower peak CI (5.0 [4.7–6.3] vs. 7.4 [7.0-8.8] L·min^-1^/m^2^, *P* = 0.004) without a significant difference in a-vO_2_diff (14.4 [11.8–16.9] vs. 13.6 [10.9–15.4] mLO_2_/dL, *P* = 0.589).

**Conclusions:**

Noninvasive measurement of peak VO_2_ Fick determinants is feasible and reliable with an ExeCMR + CPET protocol in those treated for a hematologic malignancy and may offer insight into the mechanisms of exercise intolerance in those experiencing fatigue.

**Supplementary Information:**

The online version contains supplementary material available at 10.1186/s40959-023-00182-1.

## Introduction

Exercise testing with simultaneous ventilatory expired gas-analysis (i.e., cardiopulmonary exercise testing [CPET]), provides a wealth of clinically valuable information in all populations, from those who are apparently healthy to patients diagnosed with one or more conditions. The information obtained from exercise testing, particularly information pertaining to prognosis, gauging the degree of pathophysiology present, and assessment of treatment efficacy, has prompted recognition of this assessment as a vital sign measurement [1]. An important link between exercise testing and physiology/pathophysiology is the primary dependence of aerobic capacity (i.e., peak oxygen consumption [VO_2_]) on cardiac function. Historically, accurate measurements of exercise cardiac reserve (i.e., changes in cardiac function from rest to exercise) rely upon invasive hemodynamic measurements. More recent advancements in exercise stress cardiac magnetic resonance (ExeCMR) imaging, however, allows for a reliable non-invasive alternative. Its high reproducibility has prompted some to consider it the gold-standard for assessing cardiac function during exercise [2–4]. When coupled with CPET, ExeCMR permits a comprehensive assessment of cardiorespiratory fitness (CRF), specifically aerobic capacity, and allows a mechanistic understanding thereby defining the cardiac contribution and its impact on exercise tolerance. This comprehensive assessment of exercise tolerance may have particular utility in hematologic cancer survivors who are either symptomatic or “at risk” for CVD or heart failure (HF) [5]. Of particular importance is the relatively limited amount of exercise testing research performed in hematologic cancer survivors at this time, warranting additional investigation to establish reliable and valid approaches for both clinical practice and as an endpoint measure for future research trials.

Accordingly, the goal of the current pilot study was to leverage available advanced techniques toward a combined ExeCMR + CPET protocol (evaluating its feasibility and reproducibility), and then use this methodology to discern the potential cardiovascular abnormalities that contribute to fatigue, exercise intolerance and diminished CRF in patients treated for hematologic malignancies presenting with fatigue.

## Methods

The study was approved by the VCU institutional review board and adhered to the Declarations of Helsinki.

### Feasibility

A feasibility study was performed to establish a simultaneous ExeCMR + CPET protocol that included both healthy volunteers and hematologic oncology patients with clinically-significant fatigue. Feasibility was assessed based on the ability to measure the Fick equation components of VO_2_; (1) cardiac output (CO); and (2) calculation of the arteriovenous oxygen content difference (a-vO_2_diff) with symptom-limited ExeCMR. Furthermore, we evaluated the relationship between clinical-standard upright CPET and supine exercise parameters. Criteria for a maximal ExeCMR + CPET test was assessed based on reaching a peak respiratory exchange ratio (RER) ≥ 1.00. Additionally, the ability to reach the ventilatory anaerobic threshold (VAT) and a rating of perceived exertion (RPE) ≥ 15 (6–20 scale), submaximal markers of subject effort used for exercise prescription, risk stratification, and HF prognostication, were assessed as previous ExeCMR studies have indicated reductions in traditional indices of maximal effort in the supine position when compared to standard upright exercise [6, 7]. Due to the comprehensive nature of the ExeCMR + CPET procedure, a modified version of a previously used patient acceptance questionnaire [8] was administered after completion of each procedure to determine acceptability, tolerability, and identify process improvement opportunities.

### Reproducibility

To evaluate reproducibility of the ExeCMR + CPET procedure, healthy volunteers were recruited to undergo a test-retest protocol to evaluate the reliability of peak VO_2_ and other CPET measures (minute ventilation to carbon dioxide production [VE/VCO_2_] slope; oxygen uptake efficiency slope [OUES]; partial pressure end-tidal carbon dioxide [PetCO_2_] at rest and ventilatory anaerobic threshold [VAT]), cardiac index (CI), and a-vO_2_diff measures.

### Pilot discriminatory ability

Finally, a substudy was conducted to assess the potential discriminatory power of this technique in which we compared peak VO_2_ and its Fick determinants in hematologic cancer survivors with fatigue with that of age/gender-matched healthy controls to collect pilot data for future research.

### Participants

Inclusion criteria (all subjects) consisted of middle-older age adults (35–80 years-old) who were able to exercise on a bicycle ergometer. Additional inclusion criteria for the cancer cohort consisted of a diagnosis of hematologic malignancy, prior receipt of chemotherapy, Eastern Cooperative Oncology Group status 0–2, and symptoms of clinically-significant fatigue using the National Comprehensive Cancer Network (NCCN, ≥ 4/10 on a 0–10 scale) and Functional Assessment of Chronic Illness Therapy-Fatigue scales (FACIT-F, score < 34) [9, 10].

Exclusion criteria (all subjects) consisted of contraindications to CMR or exercise testing, prior history of CVD or HF, pregnancy, or inability to give informed consent. Additional exclusion criteria for healthy controls consisted of any history of cancer, chemotherapy or radiotherapy, or any significant comorbidities or uncontrolled CVD risk factors (i.e., resting hypertension > 140/90 mmHg).

### Study design

Once enrolled, participants were scheduled for Visit-1 which included a history and physical, in part to exclude contraindications to perform exercise testing and undergo an ExeCMR protocol. Phlebotomy was performed for measurement of hemoglobin and subjects answered questionnaires related to NCCN/FACIT-F fatigue screening and physical activity levels [11]. Baseline pulmonary function testing was performed followed by a maximal symptom-limited upright CPET using a bicycle ergometer according to standard recommendations [12, 13].

Within 2-weeks from the first visit, patients were scheduled for Visit-2 (at the same time of day as Visit 1) in the ExeCMR suite. Here they completed a supine ExeCMR + CPET examination. Subjects within the healthy volunteer group who participated in the reproducibility assessment returned for a Visit-3 within 2-weeks, which again included the ExeCMR + CPET protocol.

### Cardiopulmonary exercise testing

Visit-1 CPET was performed on an upright cycle ergometer (Lode Corival, Lode BV, Netherlands) using an individualized ramping protocol between 7 and 25 watts per minute with continuous 12-lead ECG monitoring and ventilatory gas-analysis (Ultima CardioO_2_, MGC Diagnostics, Saint Paul, MN). Peak VO_2_ was recorded as the highest 30-second value obtained during the last minute of exercise and expressed in absolute values (L·min^− 1^), relative to bodyweight (mL·kg^− 1^·min^− 1^), body-surface area (BSA; L·min^− 1^/m^2^), and percent (%) of predicted values. Percent of predicted peak VO_2_ was calculated by the equations proposed by Wasserman and colleagues [14]. The VAT was calculated according to dual-methods criteria [14]. The VE/VCO_2_ slope was recorded throughout the entire exercise period [15]. The OUES was determined from the linear relation of VO_2_ versus the logarithmic transformation of VE during the entire exercise period. The PetCO_2_ was recorded in mmHg from at least two-minutes of resting data and at the value that coincided with the VAT. Blood pressure (BP) was measured using an exercise-compatible automated system (Suntech Medical, Morrisville, NC). Mean arterial pressure (MAP) was calculated as MAP= [Systolic BP + (2 x diastolic BP)/3]. The RPE [16] was assessed serially and the reason for test termination was obtained in the immediate recovery period.

Visit-2 consisted of a combined ExeCMR + CPET protocol using an MRI-compatible supine cycle ergometer (Lode MRI ergometer, Lode BV, Netherlands). Following resting image acquisition with the patient’s lower extremities positioned on the ergometer pedals, exercise was performed in a step-wise fashion with three-minute stages at 20%, 40%, 60%, and up to 80% of the peak workload obtained during the previous upright exercise test until reaching volitional fatigue or inability to maintain pedal cadence (> 50 revolutions per minute). The literature to date indicates most subjects reach volitional fatigue during supine exercise at ≈ 60% of the peak workload attained with upright exercise [2, 17]. A discontinuous incremental exercise protocol was employed (i.e., exercise was paused briefly at each 3-minute stage for rapid table repositioning/image acquisition) for CMR image acquisition.

Patients were fitted with MRI-compatible ECG, pulse-gating, pulse oximetry, and BP monitoring systems to measure heart rate (HR), oxygen saturation, and systemic BP’s (Siemens Medical Solutions USA, Malvern, PA, United States; Phillips INVIVO Expression MR400, Koninklijke Philips NV, Netherlands). Heart rate was recorded off-line and evaluated using the pulse-gating, and INVIVO HR readings. Ventilatory expired gas analysis was performed inside the scanner bore using a patient-interface breathing circuit coupled to a vendor-modified extended-length sample line that underwent successful gas and flow calibrations before every test per manufacturer recommendations.

### Cardiac magnetic resonance imaging

Imaging was performed on a Magnetom Vida 3 Tesla scanner (Siemens Healthcare, Erlangen, Germany) with prospective finger-tip pulse-gated imaging using free-breathing compressed-sensing real-time cine sequences. Pulse-gating image acquisition was utilized over ECG-gating due to the known magneto-hydrodynamic effects of MRI on the ECG signal that worsened with exercise [18]. Imaging parameters are detailed in Supplemental Table 1. Sampling was set at 1.5 cardiac cycles due to delayed acquisition with peripheral pulse gating in order to ensure a full cardiac cycle was captured for each sequence. Images were obtained at rest, each exercise stage, and following recovery. Short-axis slices were obtained from above the mitral valve through the apex immediately followed by horizontal long-axis four-chamber and two-chamber images.

Post-processing was performed using a commercially-available software program (Precession, Heart Imaging Technologies, Durham, NC). Analysis of CMR tracings were performed by a CMR core lab blinded to group assignment. The left ventricle endocardial borders were manually contoured for each short-axis slice during diastole and systole to determine the left-ventricular end-diastolic and end-systolic volumes (LVEDV, LVESV) calculated as a summation of discs [19]. Papillary muscles and trabeculations were considered as part of the blood pool. Stroke volume (SV) was measured as LVEDV minus LVESV with CO calculated as SV×HR. The left-ventricular ejection fraction (LVEF) was calculated as LVEDV-LVESV$/$LVEDV with all ventricular volumes indexed to BSA. Cardiac reserve was defined as the difference (Δ) between rest and peak exercise CO. The a-vO_2_ diff was calculated from the quotient of the VO_2_ divided by the CO according to the Fick equation.

### Statistics

Sample size for the feasibility study was based upon the ability to demonstrate a positive correlation (R > 0.80) between the upright and supine peak VO_2_ requiring at least 10 subjects to provide > 80% power with an α = 0.05. Data are presented as number (%), mean ± standard deviation (SD), or median [interquartile range, IQR] for potential deviation from a Gaussian distribution. Categorical variables were analyzed using Fisher’s exact test. Spearman’s correlations were analyzed for continuous variables. Wilcoxon signed-rank test was used to compare mean differences between upright and supine exercise on the same subject. A multivariable linear regression model (Enter method) was performed on the Fick components (CI, a-vO_2_diff) obtained at ExeCMR + CPET to determine their independent associations with peak VO_2_.

In the healthy controls group only, a general linear model using a repeated-measures analysis of variance (ANOVA) was used to assess for changes in cardiac volumes, HR, VO_2_, and a-vO_2_diff from rest through each stage of exercise. Sphericity testing was performed following repeated-measures ANOVA to determine the need for corrections. Post-hoc comparisons were performed on significantly different mean values following repeated-measures ANOVA using the Sidak method.

For the reproducibility study, intra-class correlation coefficients (ICC) with 95%CI were determined based on a mean-rating (k = 2) using a two-way mixed-effects model (absolute agreement) for reliability of peak VO_2_, CI, and a-vO_2_ diff. The reproducibility study sample size was based upon an expected ICC of ≥ 0.80 requiring at least 7 subjects. A one-sample t-test was performed to assess for significance of the mean differences between test-retest procedures. Bland Altman plots were performed to evaluate bias and limits of agreement (LOA) between the mean differences for the Fick components between test-retest studies (Supplemental Fig. 1). Linear regression was performed on the test-retest mean differences to assess for proportional bias. Additionally, an inter-rater variability analysis was determined using ICCs [95%CI] with a two-way mixed-effects model (absolute agreement) for quantification of the CMR LVEDV and LVESV by two independent readers (fully-crossed design).

For the substudy comparing peak VO_2_ and its determinants between hematologic cancer survivors with fatigue and age/gender-matched healthy volunteers a one-way ANOVA was used to assess differences between the groups. All statistical analyses were performed using SPSS v26.0 (IBM Corp, Armonk, NY) with significance set at *P* < 0.05.

## Results

### Feasibility study

The feasibility cohort included 16 total subjects (n = 10 healthy controls and n = 6 patients with a hematologic malignancy). The entire cohort was middle-age (57 [45–61] years), included seven (44%) females, predominantly Caucasian (n = 14 [88%]) with a body mass index of 26.2 [22.8–27.2] kg/m^2^ and BSA of 1.92 [1.70–2.10] m^2^. The group of hematologic cancer survivors symptomatic for fatigue consisted of patients with myelofibrosis (n = 2), acute myeloid leukemia (n = 2), non-Hodgkin’s lymphoma (n = 1), and chronic myelomonocytic leukemia (n = 1).

**Fig. 1:**
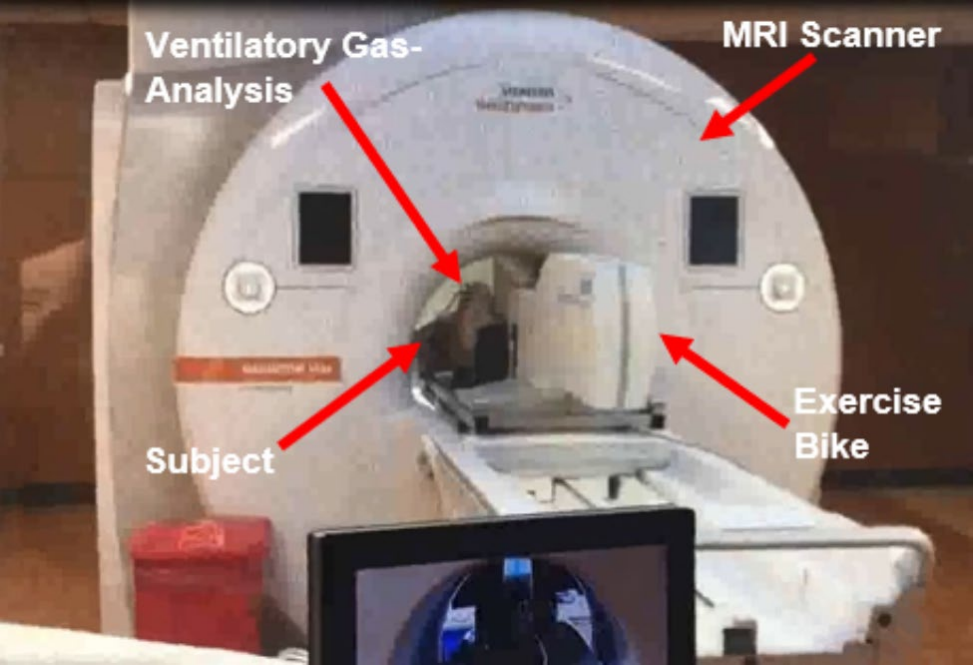
Image of a subject undergoing supine bicycle exercise stress cardiac magnetic resonance with concurrent ventilatory gas-analysis

**Fig. 2:**
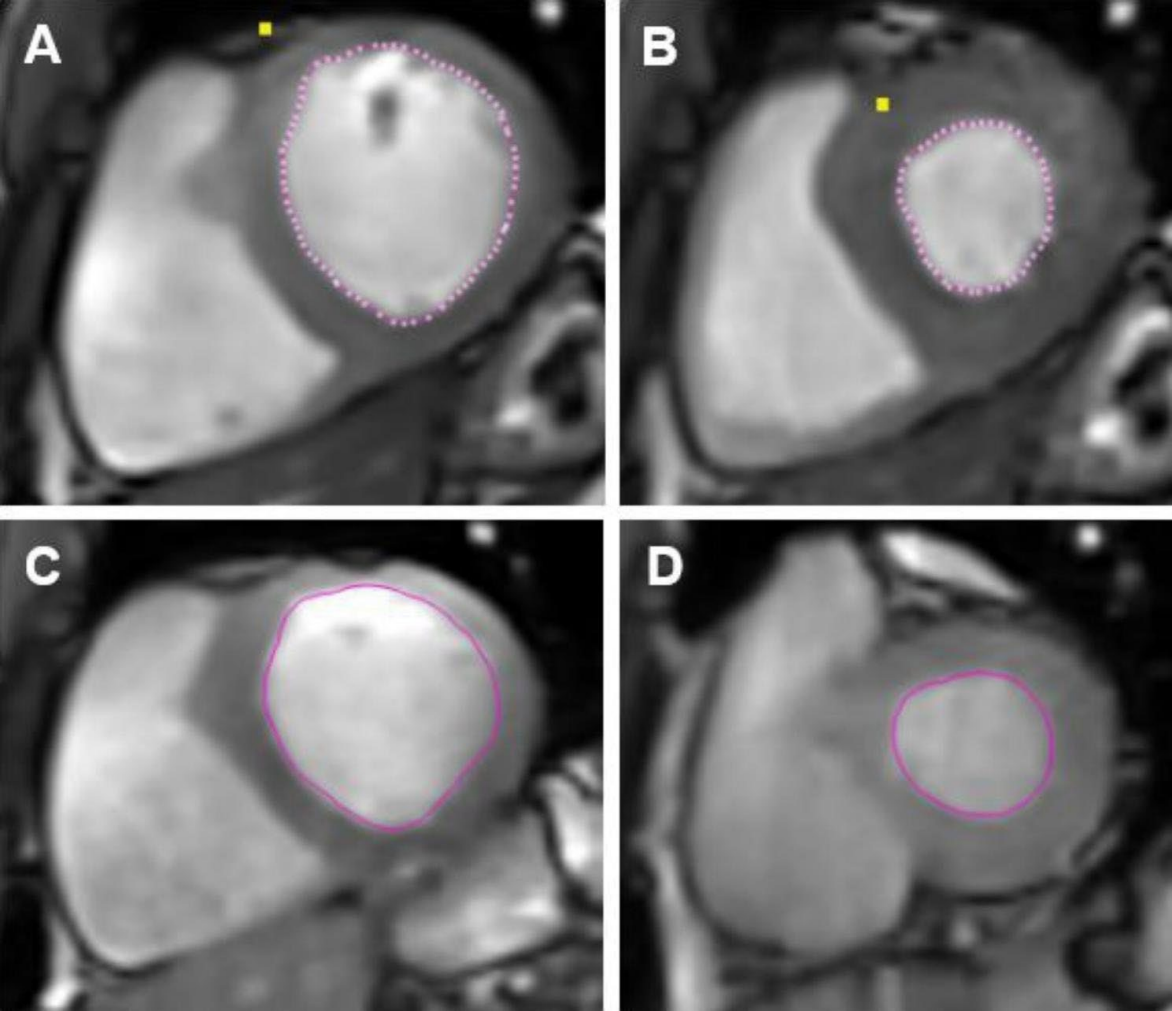
Example of basal short-axis slice images for quantification of left-ventricular function. Basal short-axis slices (Rest and Exercise) using free-breathing compressed-sensing real-time cine sequences. On each image the myocardium is gray and the cavitary blood is white. Rest (HR = 50 bpm, RR = 11 breaths/minute) **A/B**: Rest end-diastole; end-systole. Exercise (110 watts, HR = 101 bpm, RR = 21 breaths/minute) **C/D**: Exercise end-diastole; end-systole. Abbreviations: HR = heart rate; RR = respiratory rate

All 16 subjects were able to complete study procedures without any adverse events and successful collection of Fick component measurements (VO_2_, CO, a-vO_2_diff). Figures 1 and 2 illustrate a subject undergoing the ExeCMR + CPET procedure and an example of a short-axis slice at the base of the heart used to quantify LV volumes under different conditions (**Panels A,B**: rest, **Panel C,D**: exercise). The mean time from end of exercise to image acquisition was 5 [3-7] seconds with a ∆HR decrease between end of exercise and time to image acquisition of 2 [1-5] bpm. Multivariate linear regression was performed on the peak exercise CI (standardized-*β* = 0.707, *P* < 0.001) and a-vO_2_diff (standardized-*β* = 0.765, *P* < 0.001) to confirm their relationship with peak VO_2_ where both were retained as independent predictors (R^2^ = 0.89, *P* < 0.001). Table 1 demonstrates observed changes in the Fick components between rest, submaximal exercise, and peak exercise for the healthy controls. Additionally, there were significant univariate correlations between peak exercise CI and the OUES (R = 0.622, *P* = 0.031), peak oxygen pulse (R = 0.720, *P* = 0.008), rest PetCO_2_ (R = 0.797, *P* = 0.002), and PetCO_2_ at VAT (R = 0.856, *P* < 0.001) with a trend for an inverse association with the VE/VCO_2_ slope (R=-0.531, *P* = 0.075).

**Table 1:** Changes in Healthy Subject (n = 10) cardiac volumes, oxygen consumption, and calculated arteriovenous oxygen content difference with supine exercise

Variable	Workload (watts)	CMR + CPET	Mean Difference (95%CI)	*P*-value
LVEDV Index, mL/m^2^				0.09
Rest		79 ± 11		
Stage 1	40 ± 12	80 ± 11	+ 2 (-4, + 8)	
Stage 2	80 ± 24	81 ± 12	+ 4 (0, + 8)	
Stage 3	121 ± 36	76 ± 9	-2 (-10, + 7)	
Peak	126 ± 34	76 ± 10	-1 (-10, + 8)	
∆ (Peak minus Rest)		-4 ± 10		
LVESV Index, mL/m^2^				< 0.001
Rest		32 ± 9		
Stage 1	40 ± 12	30 ± 8	-2 (-9, + 5)	0.97
Stage 2	80 ± 24	24 ± 6	-6 (-13, + 1)	0.10
Stage 3	121 ± 36	21 ± 8	-12 (-19, + 3)	0.006
Peak	126 ± 34	22 ± 8	-11 (-20, + 3)	0.01
∆ (Peak minus Rest)		-10 ± 8		
SVI, mL/m^2^/beat				< 0.001
Rest		47 ± 7		
Stage 1	40 ± 12	51 ± 10	+ 4 (-6, + 14)	0.78
Stage 2	80 ± 24	57 ± 10	+ 10 (+ 2, + 19)	0.02
Stage 3	121 ± 36	55 ± 10	+ 10 (+ 6, + 14)	< 0.001
Peak	126 ± 34	54 ± 11	+ 10 (+ 6, + 15)	< 0.001
∆ (Peak minus Rest)		+ 7 ± 7		
LVEF, %				< 0.001
Rest		60 ± 8		
Stage 1	40 ± 12	63 ± 9	+ 3 (-6, + 13)	0.92
Stage 2	80 ± 24	70 ± 7	+ 9 (+ 1, + 17)	0.03
Stage 3	121 ± 36	72 ± 11	+ 14 (+ 8, + 20)	< 0.001
Peak	126 ± 34	71 ± 11	+ 14 (+ 7, + 20)	< 0.001
∆ (Peak minus Rest)		11 ± 7		
Heart rate, bpm				< 0.001
Rest		59 ± 6		
Stage 1	40 ± 12	88 ± 9	+ 29 (+ 21, + 37)	< 0.001
Stage 2	80 ± 24	106 ± 10	+ 47 (+ 36, + 57)	< 0.001
Stage 3	121 ± 36	131 ± 13	+ 72 (+ 57, + 87)	< 0.001
Peak	126 ± 34	134 ± 10	+ 75 (+ 63, + 86)	< 0.001
∆ (Peak minus Rest)		75 ± 10		
Cardiac Index, L·min^− 1^/m^2^				< 0.001
Rest		2.92 ± 0.35		
Stage 1	40 ± 12	4.78 ± 0.75	+ 1.88 (+ 0.90, + 2.87)	0.001
Stage 2	80 ± 24	6.25 ± 1.11	+ 3.46 (+ 2.27, + 4.66)	< 0.001
Stage 3	121 ± 36	7.71 ± 1.03	+ 4.98 (+ 3.94, + 6.01)	< 0.001
Peak	126 ± 34	7.84 ± 0.85	+ 5.04 (+ 4.10, + 5.99)	< 0.001
∆ (Peak minus Rest)		4.92 ± 0.774		
VO_2_, L·min^− 1^/m^2^				< 0.001
Rest		0.139 ± 0.019		
Stage 1	40 ± 12	0.434 ± 0.060	+ 0.295 (+ 0.234, + 0.356)	< 0.001
Stage 2	80 ± 24	0.670 ± 0.144	+ 0.531 (+ 0.375, + 0.687)	< 0.001
Stage 3	121 ± 36	0.966 ± 0.221	+ 0.827 (+ 0.582, + 1.071)	< 0.001
Peak	126 ± 34	0.988 ± 0.195	+ 0.849 (+ 0.634, + 1.064)	< 0.001
∆ (Peak minus Rest)		0.849 ± 0.185		
a-vO_2_ difference, mLO_2_/dL				< 0.001
Rest		4.8 ± 0.6		
Stage 1	40 ± 12	9.4 ± 1.6	+ 4.6 (+ 2.5, + 6.6)	< 0.001
Stage 2	80 ± 24	10.8 ± 1.8	+ 6.1 (+ 3.9, + 8.2)	< 0.001
Stage 3	121 ± 36	12.5 ± 1.9	+ 7.8 (+ 5.6, + 10.1)	< 0.001
Peak	126 ± 34	12.6 ± 1.9	+ 7.9 (+ 5.7, + 10.2)	< 0.001
∆ (Peak minus Rest)		7.8 ± 1.7		

Data are listed as mean ± SD. Mean difference [95% CI] calculated as difference between rest and each exercise stage.

Abbreviations: a-vO_2_ = arteriovenous oxygen; ∆=delta; LVEDV = left-ventricular end-diastolic volume; LVEF = left-ventricular ejection fraction; LVESV = left-ventricular end-systolic volume; SVI = stroke volume index; VO_2_ = oxygen consumption.

### Comparison of upright versus supine exercise

When examining the relationships between the peak upright versus supine exercise conditions there were significant strong positive associations for all expressions of VO_2_ (absolute VO_2_, R = 0.88, *P* < 0.001; relative VO_2_, R = 0.906, *P* < 0.001; BSA VO_2_, R = 0.888, *P* < 0.001), respectively. Additionally, there were significant associations for power output, VAT, VE/VCO_2_ slope, OUES, PetCO_2_ (rest, VAT), oxygen pulse, VE, respiratory rate (RR), tidal volume (V_T_), HR,, and MAP (Supplemental Table 2). However, peak VO_2_, RER, VE, RR, V_T_, and HR were significantly lower in the supine exercise condition (all *P* < 0.05).

A peak RER ≥ 1.00 at upright CPET was evidenced in 15/16 (94%) of subjects and 10/16 (63%) during supine exercise. Additionally, ability to reach/detect the VAT was evidenced in all 16 subjects during upright CPET and in 15/16 (94%) of subjects with supine exercise. Finally, 14/16 (88%) of subjects reached an RPE of ≥ 15 during the supine ExeCMR + CPET procedure.

### Patient acceptance & tolerability of procedures

Following each procedure subjects were asked to complete a patient acceptability questionnaire regarding test preparation, degree of concern, comfort, helplessness, pain (0–10 visual analog scale), willingness to repeat testing, and overall satisfaction. For all three procedures, all subjects rated the test preparation as good or better, no one rated concern more than moderate severity, acceptable comfort, degree of helplessness as ≤ moderate, pain as minimal (≤ 2/10), no one reported unwillingness to repeat tests, and overall satisfaction was acceptable with the majority rating very good or better for all procedures (Supplemental Table 3).

### Reproducibility study

Seven of the 10 healthy controls underwent a test-retest reproducibility study of the ExeCMR + CPET protocol on 2 separate visits at a median of 7 [range = 6–10] days apart. The ICC for the test-retest analysis were excellent for peak VO_2_ (ICC = 0.992, 95%CI 0.955–0.999; *P* < 0.001], good to excellent for peak CI (ICC = 0.970; 95%CI 0.838–0.995; *P* < 0.001), and good to excellent for peak a-vO_2_ diff (ICC = 0.953; 95%CI 0.744–0.992; *P* < 0.001). Linear regression analysis revealed no significant proportional bias for the test-retest mean peak VO_2_ (*P* = 0.27), CI (*P* = 0.19), and a-vO_2_ diff (*P* = 0.74). Table 2 shows the mean difference, standard deviation of the difference, standard error of measure, smallest detectable change (SDC) of an individual and group, and the 95% LOA for peak VO_2_, CI, SVI, the calculated a-vO_2_ diff, and other CRF variables. Based upon the SDC for the group, a change in relative VO_2_ of 0.9 mL·kg^− 1^·min^− 1^ or 4% would need to occur to detect a significant change following an intervention. Likewise, for peak exercise CI, a change of 0.24 L·min^− 1^/m^2^ or 3% would be required to detect a significant change following an intervention. Given the variance of our method, 29 subjects would need to be enrolled in each arm of a randomized clinical trial to detect significant differences in exercise associated cardiac output with 80% power. Finally, inter-rater reliability was assessed for the quantification of left-ventricular volumes (LVEDV, LVESV) during exercise in the reproducibility study cohort revealing moderate to excellent ICC’s for LVEDV (ICC = 0.912; 95%CI 0.550–0.985; *P* = 0.005) and LVESV (ICC = 0.948; 95%CI 0.651–0.991; *P* < 0.001), respectively.

**Table 2:** Comparison of Test-Retest ExeCMR + CPET Peak Exercise Values in Reproducibility Healthy Cohort

Fick Variables	Test	Retest	_mean_diff	SD_diff_	SEM	SDC_ind_	SDC_group_	95%LOA
BSA VO_2_,								
L·min^− 1^/m^2^	0.984 ± 0.235	0.981 ± 0.240	0.003	0.045	0.032	0.088	0.033	-0.085, 0.091
Relative VO_2_,								
mL·kg^− 1^·min^− 1^	24.9 ± 4.8	24.8 ± 5.08	0.114	1.162	0.822	2.3	0.9	-2.2, 2.3
Cardiac Index,								
L·min^− 1^/m^2^	7.96 ± 0.966	8.04 ± 0.888	0.082	0.318	0.225	0.62	0.24	-0.71, 0.54
SVI, mL/m^2^/beat	59.8 ± 7.1	60.4 ± 8.0	0.533	2.163	1.530	4.2	1.6	-4.8, 3.7
a-vO_2_ difference,								
mLO_2_/dL	12.28 ± 2.06	12.08 ± 1.99	0.200	0.892	0.631	1.75	0.66	-1.55, 1.95
**Other CPET Variables**								
VE/VCO_2_ slope	27.4 ± 2.6	28.2 ± 2.1	0.74	2.077	1.468	4.07	1.54	-3.33, 4.07
OUES	1.91 ± 0.6	1.91 ± 0.6	0.003	0.144	0.102	0.28	0.11	-0.28, 0.28
Rest PetCO_2_	38.0 ± 4.9	37.5 ± 4.1	0.49	2.889	2.042	5.7	2.14	-5.18, 6.15
PetCO_2_ at VAT	43.0 ± 3.4	42.3 ± 3.0	0.71	3.904	2.760	7.6	2.89	-6.94, 8.37

Data are expressed as mean ± SD.

Abbreviations: a-vO_2_ = arteriovenous oxygen; BSA = body surface area; ExeCMR + CPET = exercise cardiac magnetic resonance + cardiopulmonary exercise test; LOA = limits of agreement; _mean_diff=mean test-retest difference; OUES = oxygen uptake efficiency slope; PetCO_2_ = partial pressure end-tidal carbon dioxide; SD_diff_=standard deviation of the difference; SDC_group_= smallest detectable change of group; SDC_ind_=smallest detectable change of individual; SEM = standard error of measurement; SVI = stroke volume index; VAT = ventilatory anaerobic threshold; VE/VCO_2_ = minute ventilation to carbon dioxide production; VO_2_ = oxygen consumption.

### Substudy comparing hematologic cancer survivors with fatigue and age/gender matched healthy controls

Table 3 demonstrates the baseline characteristics of the six patients with a hematologic malignancy compared with age and gender-matched healthy controls. The Karnofsky performance status was lower in subjects with cancer while the HCT-specific comorbidity index was higher subjects compared with healthy controls. Additionally, the cancer subjects had significantly higher reports of fatigue as per study design. Hemoglobin and physical activity levels were not significantly different between the groups. Table 4 describes the upright cycle ergometer CPET results and the comparisons between groups. Pre-exercise spirometry values were all within normal diagnostic limits and not significantly different between the groups. The cancer group demonstrated a numerically lower power output (Watts) and exercise time and significantly lower peak VO_2_ values. Importantly, objective and subjective indicators of subject effort including the peak RER, peak HR, and peak RPE were not significantly different between groups.

**Table 3:** Baseline Characteristics comparing patients with hematologic malignancies to matched-healthy Controls

Variable	Healthy Controls (n = 6)	Patients with Cancer (n = 6)		*P*-value
Age, years	58 [43–64]	59 [49–63]		0.818
Sex				1.000
Female	2 (33%)	2 (33%)		
Male	4 (67%)	4 (67%)		
Race				0.455
Caucasian	6 (100%)	4 (67%)		
African-American	0 (0%)	2 (33%)		
Weight, kg	75.9 [70.4–89.6]	86.7 [76.0–95.0]		0.240
BMI, kg/m^2^	27.0 [25.0-27.2]	26.9 [25.5–30.4]		0.485
BSA, m^2^	1.86 [1.79–2.12]	2.07 [1.88–2.13]		0.589
Karnofsky Status, %	100 [100–100]	90 [80–90]		**0.024**
HCT-Comorbidity Index	0 [0–0]	3.5 [2.8-5.0]		**0.024**
Hemoglobin, g/dL	14.3 [13.0-14.8]	12.7 [8.7–14.2]		0.247
NCCN Fatigue scale, 0–10	0 [0–0]	5.5 [4.4-7.0]		**0.024**
FACIT-F Fatigue Scale	52.0 [50.3–52.0]	21.0 [19.0-38.3]		**0.002**
IPAQ, MET/min/week	2333 [780–3942]	1184 [248–1873]		0.240

Data are presented as median [interquartile range] or number (%). **Bold** values indicate *P* < 0.05.

Abbreviations: BMI = body mass index; BSA = body surface area; FACIT-F = Functional Assessment of Chronic Illness Therapy-Fatigue; HCT = hematopoietic cell transplant; IPAQ = International Physical Activity Questionnaire; MET/min/week = metabolic equivalents of task per minute per week; NCCN = National Comprehensive Cancer Network.

**Table 4:** Upright Cycle Ergometer Cardiopulmonary Exercise Testing

Variable	Healthy Controls (n = 6)	Patients with Cancer (n = 6)	P-value
**Pulmonary Function Results**			
FVC (%)	102 [94–107]	87 [83–107]	0.247
FEV_1_ (%)	105 [99–114]	102 [86–114]	0.792
FEV_1_/FVC ratio	0.78 [0.73–0.81]	0.79 [0.76–0.85]	0.429
**Upright Exercise Test Results**			
Power output (watts)	232 [128–268]	127 [94–149]	0.065
Exercise Time (sec)	706 [610–806]	526 [450–600]	0.093
Work Efficiency (mL/min/Watt)	8.8 [8.8–9.7]	8.0 [6.1–10.2]	0.394
Peak VO_2_ (mL·kg^− 1^·min^− 1^)	31.7 [21.7–33.8]	17.5 [11.7–19.5]	**0.009**
%-predicted peak VO2	107 [100–112]	61 [47–79]	**0.002**
Peak VO_2_ (L·min^− 1^/m^2^)	1.30 [0.82–1.42]	0.70 [0.50–0.88]	**0.015**
Ventilatory Anaerobic Threshold (mL·kg^− 1^·min^− 1^)	16.4 [14.0-22.3]	9.9 [8.1–13.8]	**0.015**
Peak RER	1.18 [1.13–1.33]	1.22 [1.14–1.28]	0.818
VE/VCO_2_ slope	26.6 [23.5–29.9]	30.2 [27.4–39.7]	0.132
OUES	2.47 [1.55–2.89]	1.69 [1.15–2.07]	0.240
Rest PetCO_2_, mmHg	34.8 [32.1–38.9]	29.0 [25.5–34.1]	0.093
PetCO_2_ at VAT, mmHg	42.0 [38.8–47.3]	36.0 [30.8–38.0]	**0.009**
Resting HR (bpm)	64 [57–69]	85 [71–93]	**0.041**
Peak HR (bpm)	143 [132–164]	143 [125–157]	0.394
%-predicted peak HR	90 [84–95]	87 [74–96]	0.818
Rest MAP, mmHg	92 [86–102]	84 [75–86]	**0.015**
Peak MAP, mmHg	114 [105–123]	99 [87–106]	**0.041**
RPE (6–20)	17.0 [16.8–18.5]	16.5 [14.8–19.0]	0.589
Dyspnea (0–10)	4.5 [3.8–7.3]	4.0 [3.8–8.3]	1.000

Data are presented as median [interquartile range] or number (%). P-values for difference between cancer patients and healthy controls. **Bold** values indicate *P* < 0.05.

Abbreviations: BSA = body surface area; FVC = forced vital capacity; FEV_1_ = forced expiratory volume 1-second; HR = heart rate; MAP = mean arterial pressure; OUES = oxygen uptake efficiency slope; PetCO_2_ = partial pressure end-tidal carbon dioxide; RER = respiratory exchange ratio; RPE = rating of perceived exertion; VAT = ventilatory anaerobic threshold; VE/VCO_2_ = minute ventilation to carbon dioxide production; VO_2_ = oxygen consumption.

**Fig. 3:**
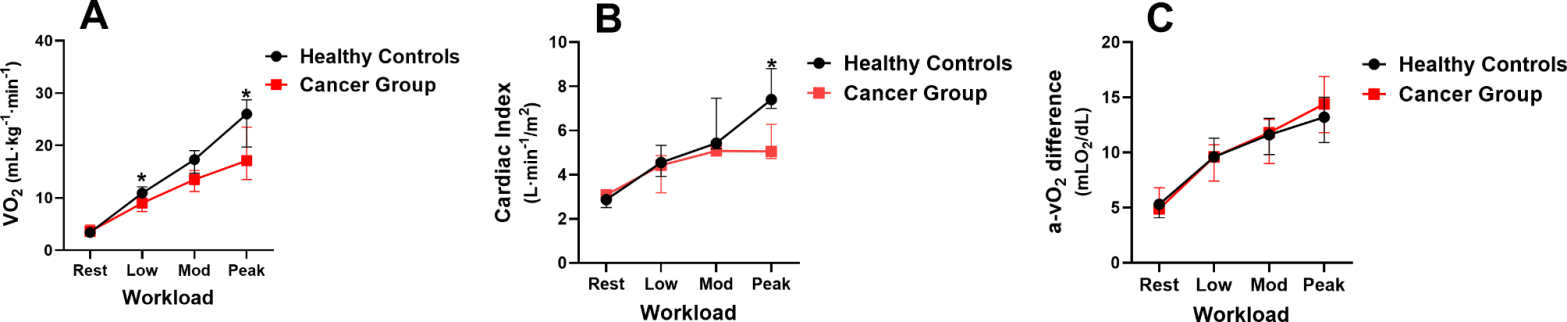
Change in Fick components during supine ExeCMR + CPET between Healthy Controls and Cancer Patients. **A:** VO_2_, **B:** Cardiac Index, C: a-vO_2_ diff. Each panel displays values at rest, low and moderate intensity, and peak exercise. *p < 0.05 between healthy controls and cancer group. Abbreviations: a-vO_2_ = arteriovenous oxygen difference; CPET = cardiopulmonary exercise test; VO_2_ = oxygen consumption

Table 5 details the combined supine ExeCMR + CPET parameters and group comparisons. The LV mass index and resting LVEDVI were not significantly different between the groups. The resting LVESVI was significantly higher while the SVI and LVEF were significantly lower in the cancer group although the resting CI was not significantly different due to the cancer groups higher resting HR. Resting relative VO_2_, a-vO_2_diff, RER, and MAP values were not significantly different between groups. During supine peak exercise the SVI, LVEF, CI, and VO_2_ were significantly lower in cancer subjects. However, peak LVEDVI, HR, RER, RPE, MAP, and a-vO_2_diff were not significantly different between groups (Fig. 3).

**Table 5:** Supine ExeCMR + CPET Parameters

Variable	Healthy Controls (n = 6)	Patients with Cancer (n = 6)	*P*-value	
**Rest Parameters**				
LV Mass Index, grams/m^2^	69.7 [63.4–75.7]	64.1 [59.4–71.8]	0.485	
LVEDV Index, mL/m^2^	75.3 [71.0-93.2]	84.0 [74.5–86.6]	0.818	
LVESV Index, mL/m^2^	29.6 [23.5–38.2]	40.0 [32.6–44.0]	**0.041**	
SVI, mL/m^2^/beat	47.6 [45.4–55.0]	40.5 [38.7–46.3]	**0.041**	
LVEF, %	60.5 [58.8–67.3]	51.0 [47.8–55.8]	**0.026**	
Cardiac Index, L·min^− 1^/m^2^	2.87 [2.51–3.03]	3.09 [2.80–3.29]	0.180	
HR, bpm	56 [52–62]	70 [63–85]	**0.009**	
MAP, mmHg	89 [84–102]	87 [82–91]	0.394	
VO_2_, L·min^− 1^/m^2^	0.133 [0.118–0.158]	0.156 [0.136–0.204]	0.132	
VO_2_, mL·kg^− 1^·min^− 1^	3.4 [2.9–3.8]	3.6 [3.2–4.8]	0.310	
RER	0.85 [0.77–0.92]	0.82 [0.74–0.95]	0.699	
a-vO_2_ difference, mLO_2_/dL	5.3 [4.1–5.4]	4.9 [4.7–6.8]	0.818	
VE, L·min^− 1^	7.1 [5.9–8.4]	9.3 [7.6–12.9]	**0.026**	
RR, breaths/min	11 [11-14]	16 [14-21]	**0.015**	
V_T_, L	0.61 [0.54–0.63]	0.57 [0.43–0.74]	1.000	
**Peak Exercise Parameters**				
Power output, Watts	147 [96–165]	95 [61–105]	0.065	
LVEDV Index, mL/m^2^	75.9 [72.1–85.1]	72.8 [65.3–81.4]	0.589	
LVESV Index, mL/m^2^	20.5 [13.5–22.3]	29.3 [19.7–36.4]	0.132	
SV Index, mL/m^2^	60.2 [53.7–64.6]	42.4 [36.8–50.3]	**0.026**	
LVEF, %	76.5 [71.3–81.5]	59.5 [53.3–72.8]	**0.015**	
Cardiac Index, L·min^− 1^/m^2^	7.4 [7.0-8.8]	5.0 [4.7–6.3]	**0.004**	
HR, bpm	129 [124–136]	124 [105–140]	0.485	
MAP, mmHg	117 [100–127]	117 [102–133]	0.931	
VO_2_, L·min^− 1^/m^2^	1.07 [0.74–1.25]	0.73 [0.58-1.00]	0.065	
VO_2_, mL·kg^− 1^·min^− 1^	26.0 [19.7–29.5]	17.1 [13.5–23.5]	**0.026**	
RER	1.05 [1.01–1.09]	0.98 [0.91–1.26]	0.485	
a-vO_2_ difference, mLO_2_/dL	13.6 [10.9–15.4]	14.4 [11.8–16.9]	0.589	
VE, L·min^− 1^	61.9 [40.3–80.0]	53.2 [34.9–61.7]	0.310	
RR, breaths/min	31 [26-35]	32 [27-37]	0.818	
V_T_, L	2.00 [1.73–2.28]	1.78 [1.18–2.03]	0.240	
RPE (6–20)	16 [15-17]	17 [16-18]	0.589	

Data are presented as median [interquartile range]. *P*-values for difference between patients with cancer and healthy controls.

Abbreviations: a-vO_2_ = arteriovenous oxygen; CMR + CPET = exercise stress cardiac magnetic resonance coupled with cardiopulmonary exercise testing; HR = heart rate; LV = left-ventricle; LVEDV = left-ventricular end-diastolic volume; LVEF = left-ventricular ejection fraction; LVESV = left-ventricular end-systolic volume; MAP = mean arterial pressure; RER = respiratory exchange ratio; RR = respiratory rate; SV = stroke volume; VE = minute ventilation; VO_2_ = oxygen consumption; V_T_=tidal volume.

## Discussion

In this pilot feasibility study, we determined that a technique employing simultaneous measures from exercise-associated CMR and CPET to symptom-limited peak exertion was feasible in both middle-aged healthy controls and hematologic cancer survivors with fatigue. Furthermore, we demonstrated high reproducibility of this combined technique and determined the SDC required when using this technique to assess the potential efficacy of an intervention. Finally, we developed pilot data demonstrating its potential discriminatory ability in hematologic cancer survivors symptomatic for fatigue reflecting an impairment in exercise cardiac reserve capacity compared with age/gender-matched healthy controls potentially explaining the etiology of their fatigue, exercise intolerance, and predominant reduction in peak VO_2_.

The ability to simultaneously assess exercise cardiac reserve relative to metabolic demands (i.e., VO_2_) noninvasively has significant potential to discern the causes of exercise intolerance in patients with both normal and abnormal resting cardiac function. It has been long known that resting measures of LV function (i.e., LVEF) demonstrate a poor relationship with exercise capacity [20] although the close relationship between CO and VO_2_ is well-established, leading to the widespread use of VO_2_ as an indirect measure of cardiac reserve. Recent investigations of patients with HF syndromes (particularly those with preserved LV ejection fraction) have identified both cardiac and extra-cardiac causes of exercise intolerance leading to calls for exercise-based phenotyping [21–23]. Similarly, hematologic cancer survivors exposed to potentially cardiotoxic treatments frequently experience HF symptoms that can be difficult to characterize but are nonetheless associated with poor functional status [10, 24–26]. Ness and colleagues demonstrated exercise intolerance was associated with all-cause mortality in adult childhood cancer survivors related to treatment exposures and was associated with multi-organ system impairments including cardiac, pulmonary, autonomic, and musculoskeletal deficits [27]. This supports the notion that comprehensive assessment of CRF is necessary to identify the specific impairments in affected organ systems when considering interventions to improve functional status [28–31].

The utility of a combined and simultaneous ExeCMR + CPET technique is that is allows a non-invasive yet comprehensive assessment of the central (O_2_ delivery) and peripheral (O_2_ utilization) components of CRF that have typically only been available with invasive CPET. This combined ExeCMR + CPET technique has previously been piloted in healthy adults and applied in children with pulmonary arterial hypertension, repaired tetralogy of Fallot, and healthy controls [8, 32].

Our findings of high reproducibility for CO measurements with exercise-associated MRI have previously been confirmed [2, 4, 33, 34]. The current study adds to this body of literature by simultaneous measurement of metabolic work (i.e., VO_2_) thereby allowing a more precise quantification of the a-vO_2_ difference and by establishing the smallest detectable change required to establish efficacy of a therapeutic intervention or clinical change when considering measurement variability. Indeed, for peak exercise CI, we found a smallest detectable group change of 0.24 L·min^− 1^/m^2^ or 3% would be required to establish a change that exceeds measurement variability. This is in line with the findings of Dillon et al. who utilized an ExeCMR technique to evaluate changes in cardiac function that occurred in hematological cancer patients undergoing allogeneic hematopoietic stem cell transplant (allo-HCT) [35]. In their study, allo-HCT patients experienced a -1.0 (95%CI: -1.5, -0.5) L·min^− 1^/m^2^ or 13% reduction in peak exercise CI three months following transplant compared with pre-transplant values while the Δ peak exercise CI for age-matched non-cancer controls was unchanged (-0.2 [95%CI: -0.8, 0.2] L·min^− 1^/m^2^, 2% change) demonstrating the ability to detect a clinical significant change.

Using ExeCMR to detect determinants of exercise intolerance, in pediatric cancer survivors treated with anthracycline-based chemotherapy (AC) and/or radiotherapy, Foulkes et al. identified a phenotype wherein those with reduced exercise capacity at CPET demonstrated a reduced cardiac reserve (mediated by reductions in ∆ and peak exercise CI and SVI) during subsequent supine-cycle ExeCMR compared to those with preserved exercise capacity that was not explained by resting measures of cardiac function [36]. In the formative BREXIT study, adult women with stage I-III breast cancer receiving AC therapy randomized to a 12-month exercise training intervention or usual care underwent ExeCMR before and after intervention to quantify cardiac reserve and its relationship with CRF [37]. Results showed a 1.22 (0.78, 1.67) and 1.62 (1.14, 2.09) L·min^− 1^ increase in peak exercise CO at four months and 12-months, respectively in the exercise training arm compared with reductions of -0.99 (-1.48, -0.51) and − 1.32 (-1.87, -0.76) L·min^− 1^ at four months and 12-months, respectively in the usual care group. Importantly, changes in peak VO_2_ from baseline to 12-months were significantly associated with the change in peak exercise CO (β: 0.76) illustrating the ability of ExeCMR to provide a mechanistic link to exercise training intervention improvements in CRF or the decline experienced in the AC therapy group receiving usual care.

We also demonstrated significant associations and trends between broader measures of CRF (VE/VCO_2_ slope, OUES, PetCO_2_ [rest, VAT]) and peak exercise cardiac index. These are parameters obtained during standard clinical CPET that have prognostic significance in the HF population [15] although their role in the hematologic cancer patient has yet to be elucidated. Additional research assessing the value of these CPET measures in this patient population is warranted.

### Study limitations

The current feasibility study was designed to develop an ExeCMR + CPET protocol and to assess the reproducibility of this procedure, therefore, it was not powered to detect group differences. The finding of reduced exercise tolerance due to the observance of an attenuated cardiac reserve in symptomatic hematologic cancer subjects should not be viewed as conclusive due to the low number of subjects, and while it is consistent with the cardio-oncology literature to date and hypothesis-generating it requires further study with a larger population in a longitudinal design. The finding of reduced baseline cardiac function in the hematologic cancer survivors was somewhat unexpected due to the study exclusion criteria of overt CVD/HF and may have influenced our findings.

## Conclusions

Noninvasive measurement of peak exercise VO_2_ Fick determinants is feasible and reliable with a simultaneous ExeCMR + CPET protocol, including survivors of hematologic cancer with fatigue. Preliminary evidence suggests an ExeCMR + CPET protocol can discern the cardiac contribution of exercise intolerance in hematologic cancer survivors that may be in part driven by reductions in exercise-associated cardiac reserve. This technique may have utility in at-risk patients or those with nonspecific symptoms out of proportion to resting diagnostic measurements or when more than one contributing factor may be involved.

## Electronic supplementary material

Below is the link to the electronic supplementary material.

Supplementary Material 1

Supplementary Material 2

Supplementary Material 3

Supplementary Material 4

## Data Availability

The dataset analyzed during the current study are available from the corresponding author upon reasonable request.

## Abbreviations

a-vO_2_diff: Arteriovenous oxygen difference
BP: Blood pressure
BSA: Body surface area
BMI: Body mass index
CI: Cardiac index
CO: Cardiac output
CPET: Cardiopulmonary exercise test
CVD: Cardiovascular disease
ECG: Electrocardiogram
ExeCMR: Exercise cardiac magnetic resonance
HR: Heart rate
HF: Heart failure
LVEDV: Left-ventricular end-diastolic volume
LVEF: Left-ventricular ejection fraction
LVESV: Left-ventricular end-systolic volume
MAP: Mean arterial pressure
RPE: Rating of perceived exertion
SVI: Stroke volume index
VAT: Ventilatory anaerobic threshold
VE/VCO_2_: Minute ventilation to carbon dioxide production
VO_2_: Oxygen consumption
