# Supplementary material for: Simultaneous exercise stress cardiac magnetic resonance and cardiopulmonary exercise testing to elucidate the Fick components of aerobic exercise capacity: a feasibility and reproducibility study and pilot study in hematologic cancer survivors
Source: Cardiooncology. 2023 Jul 10;9:31. doi: 10.1186/s40959-023-00182-1 (PMC10331991; doi:10.1186/s40959-023-00182-1)
Supplement: Supplementary file 1 — Supplementary Material 1 [file 40959_2023_182_MOESM1_ESM.docx]

**Supplemental Figure 1**. Bland-Altman Plots of peak VO_2_, peak Cardiac Index, peak a-vO_2_diff.


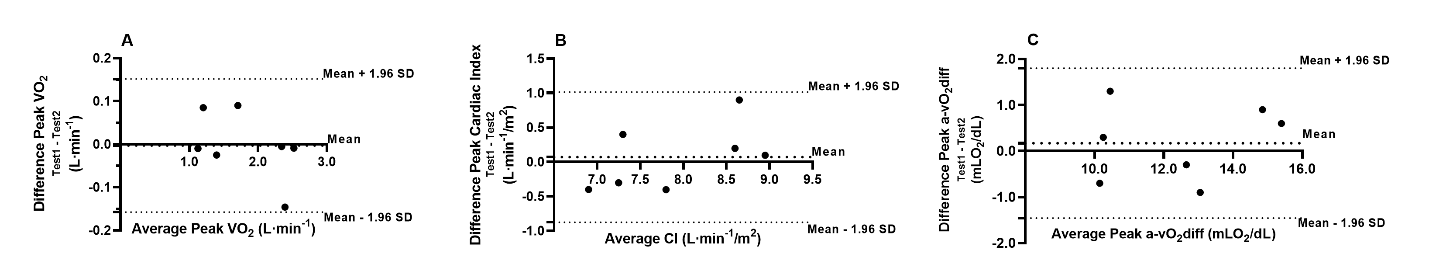


**Supplemental Figure Legend:** Mean difference between test-retest measurements of peak: A) VO_2_, B) Cardiac Index, and C) a-vO_2_diff.

**Abbreviations:** VO_2_=oxygen consumption; a-vO_2_diff=arteriovenous oxygen content difference.
