# Supplementary material for: Simultaneous exercise stress cardiac magnetic resonance and cardiopulmonary exercise testing to elucidate the Fick components of aerobic exercise capacity: a feasibility and reproducibility study and pilot study in hematologic cancer survivors
Source: Cardiooncology. 2023 Jul 10;9:31. doi: 10.1186/s40959-023-00182-1 (PMC10331991; doi:10.1186/s40959-023-00182-1)
Supplement: Supplementary file 2 — Supplementary Material 2 [file 40959_2023_182_MOESM2_ESM.docx]

| **Supplemental Table 1.** Compressed-sensing cine CMR imaging parameters. | |
| --- | --- |
| Temporal Resolution, ms | 29.26 |
| Repetition time, ms | 29.26 |
| Echo Time, ms | 1.11 |
| Flip angle, degree | 80 |
| Field of view, mm | ~380 |
| Acquisition matrix | 192/ 75% (101/ 192) |
| Slice thickness, mm | 6 |
| Spatial resolution, mm | 2 x 2 x 6 (2.6 x 2 x 6) |
| Bandwidth, Hz/pixel | 1042 |
| Acceleration factor | 9.2 T-PAT |
| Image Filter Settings |  |
| Intensity | Medium |
| Edge Enhancement | 4 |
| Smoothing | 2 |
| Abbreviations: ms=milliseconds; mm=millimeters; Hz=Hertz;  T-PAT=Temporal Parallel Acquisition Technique. | |
