# Supplementary material for: Simultaneous exercise stress cardiac magnetic resonance and cardiopulmonary exercise testing to elucidate the Fick components of aerobic exercise capacity: a feasibility and reproducibility study and pilot study in hematologic cancer survivors
Source: Cardiooncology. 2023 Jul 10;9:31. doi: 10.1186/s40959-023-00182-1 (PMC10331991; doi:10.1186/s40959-023-00182-1)
Supplement: Supplementary file 3 — Supplementary Material 3 [file 40959_2023_182_MOESM3_ESM.docx]

| **Supplemental Table 2**. Comparison of Upright versus Supine Cycle Ergometer Exercise. | | | | | |
| --- | --- | --- | --- | --- | --- |
| Variable | Upright | Supine | % of Upright | R-value | *P*-value |
| Power output, watts | 150 [126-241] | 112 [83-148] | 61 [60-77]***** | 0.894 | <0.001 |
| Peak VO_2_, L∙min^-1^ | 1.799 [1.390-2.664] | 1.674 [1.386-2.248] | 88 [82-97]* | 0.880 | <0.001 |
| Peak VO_2_, mL·kg^-1^·min^-1^ | 26.6 [17.5-32.4] | 23.9 [17.4-27.6] | 88 [83-98]***** | 0.906 | <0.001 |
| Peak VO_2_, L∙min^-1^/m^2^ | 0.995 [0.728-1.341] | 0.953 [0.734-1.076] | 88 [82-97]* | 0.888 | <0.001 |
| Absolute VAT, L∙min^-1^ | 1.078 [0.823-1.570] | 1.180 [0.920-1.400] | 93 [86-102] | 0.811 | <0.001 |
| RER | 1.20 [1.14-1.30] | 1.03 [0.93-1.14] | 80 [77-96]***** | 0.273 | 0.307 |
| VE/VCO_2_ slope | 27.9 [25.2-30.7] | 27.4 [25.5-30.5] | 99 [89-106] | 0.546 | 0.029 |
| OUES | 1.80 [1.49-2.76] | 1.91 [1.39-2.46] | 93 [86-102] | 0.867 | <0.001 |
| Rest PetCO_2_, mmHg | 33.3 [27.7-37.0] | 38.8 [35.7-40.4] | 116 [105-127]* | 0.669 | 0.017 |
| PetCO_2_ at VAT, mmHg | 38.5 [36.0-42.0] | 41.5 [38.0-44.8] | 103 [97-115] | 0.748 | 0.005 |
| Peak Oxygen Pulse, mL/beat | 12.6 [9.0-17.6] | 13.2 [10.4-16.5] | 100 [92-112] | 0.888 | <0.001 |
| Peak VE, L∙min^-1^ | 69.6 [53.7-91.1] | 53.2 [38.4-65.8] | 74 [58-85]***** | 0.768 | <0.001 |
| Peak RR, breaths/min | 36 [29-38] | 28 [26-35] | 90 [84-97]***** | 0.568 | 0.022 |
| Peak Tidal volume, L | 2.130 [1.840-2.740] | 1.873 [1.332-2.149] | 83 [71-89]***** | 0.856 | <0.001 |
| Peak HR, bpm | 156 [131-162] | 129 [122-141] | 86 [82-94]***** | 0.764 | <0.001 |
| Peak MAP, mmHg | 103 [95-114] | 111 [99-121] | 106 [99-120] | 0.733 | 0.001 |
| Data are presented as median [interquartile range]. ******P*<0.05 for difference between upright and supine conditions.  Abbreviations: HR=heart rate; MAP= mean arterial pressure; OUES=oxygen uptake efficiency slope; PetCO_2_=partial pressure end-tidal carbon dioxide; RER=respiratory exchange ratio; RR=respiratory rate; VAT=ventilatory anaerobic threshold; VE/VCO_2_= minute ventilation to carbon dioxide production; VO_2_= oxygen consumption. | | | | | |
