# Supplementary material for: Simultaneous exercise stress cardiac magnetic resonance and cardiopulmonary exercise testing to elucidate the Fick components of aerobic exercise capacity: a feasibility and reproducibility study and pilot study in hematologic cancer survivors
Source: Cardiooncology. 2023 Jul 10;9:31. doi: 10.1186/s40959-023-00182-1 (PMC10331991; doi:10.1186/s40959-023-00182-1)
Supplement: Supplementary file 4 — Supplementary Material 4 [file 40959_2023_182_MOESM4_ESM.docx]

| **Supplemental Table 3.** Patient Acceptance Questionnaire | | | |
| --- | --- | --- | --- |
|  | Upright CPET | Rest MRI | Supine CPET+MRI |
| Preparation | 1 [1-1] | 1 [1-1] | 1 [1-2] |
| Concern | 1 [1-2] | 1 [1-1] | 1 [1-1] |
| Comfort | 2 [1-2] | 2 [1-2] | 2 [2-3] |
| Helplessness | 1 [1-1] | 1 [1-2] | 1 [1-2] |
| Pain | 0 [0-1] | 0 [0-1] | 0 [0-3] |
| Willingness to repeat, Yes | 15 (94%) | 15 (94%) | 15 (94%) |
| Overall Satisfaction | 1 [1-1] | 1 [1-2] | 1 [1-2] |
| Data are presented as median [IQR]. Preparation, Concern, Comfort, Helplessness, Overall satisfaction - graded on a 1-5 Likert scale (1=best rating, 5=worst rating). Pain - rated on a 0-10 ratio scale. Willingness to repeat treated as categorical variable. | | | |
